# Per-cell histone acetylation is associated with terminal differentiation in human T cells

**DOI:** 10.1186/s13148-024-01634-w

**Published:** 2024-02-06

**Authors:** Cheng Yang, You Li, Yaqiu Hu, Qian Li, Yinghua Lan, Yongguo Li

**Affiliations:** 1 Department of Infectious Diseases, The First Affiliated Hospital of Chongqing Medical University, Chongqing, 400016 China; 2 Department of Infectious Diseases, Key Laboratory of Molecular Biology for Infectious Diseases (Ministry of Education), Institute for Viral Hepatitis, The Second Affiliated Hospital, Chongqing Medical University, Chongqing, 400010 China

**Keywords:** Single-cell histone acetylation, Human T cell, TCF-1, Stemness, Terminal differentiation, C646

## Abstract

**Background:**

Epigenetic remodeling at effector gene loci has been reported to be critical in regulating T cell differentiation and function. However, efforts to investigate underlying epigenetic mechanisms that control T cell behaviors have been largely hindered by very limited experimental tools, especially in humans.

**Results:**

In this study, we employed a flow cytometric assay to analyze histone acetylation at single-cell level in human T cells. The data showed that histone acetylation was increased during T cell activation. Among T cell subsets, terminally differentiated effector memory T (T_EMRA_) cells robustly producing effector cytokines were hyper-acetylated. Conversely, these T_EMRA_ cells had lower expression levels of TCF-1, a key transcription factor for maintaining stem cell features. Pharmaceutical inhibition of histone acetylation using a small molecule C646 restrained the production of effector molecules, but retained stem cell-like properties in T cells after expansion.

**Conclusions:**

Per-cell histone acetylation is associated with terminal differentiation and poor stemness in human T cells. These observations suggest a new approach to enhance the stem cell-like properties of T cells and improve the efficacy of immunotherapy.

**Supplementary Information:**

The online version contains supplementary material available at 10.1186/s13148-024-01634-w.

## Introduction

Human peripheral T cell is a heterogeneous population, which comprises naïve (T_N_), effector (T_E_) and memory (T_M_) cell subsets [1]. Upon encountering exogenous and sometimes endogenous antigens, T_N_ cells become activated, undergo extensive expansion and give rise to T_E_ cells. After antigen clearance and resolution of inflammation, T_E_ cells will contract, remaining a small fraction of T_E_ cells to further differentiate into T_M_ cells [2]. A hallmark feature of T_M_ cells is long-term persistence through self-renewal, which is typically observed in stem cells [3]. T_M_ cells are conventionally divided into two subsets with differential stem cell-like properties: central memory T cells (T_CM_) represent the subset with stem cell features such as self-renewal for long periods, whereas effector memory T cells (T_EM_) are committed to terminal differentiation and lose stem cell-like features after a few rounds of divisions [4]. In humans, but not in mice, there is another CD45RA-expressing T_EM_ subset (T_EMRA_), which is also characterized by potent effector function but poor survival [5]. More recently, a novel population of T_M_ cells, defined as stem cell memory T cells (T_SCM_), was identified in humans. In comparison with conventional T_M_ cells, T_SCM_ cells display enhanced self-renewal and confer superior protection against pathogens and tumors [6]. The molecular mechanisms that direct the differentiation of aforementioned T cell subsets have not been well elucidated, especially in humans.

The process of lineage differentiation to establish cellular identity is often accompanied by epigenetic remodeling, which is well described in pluripotent stem cells [7]. Epigenetic modifications, such as DNA methylation and histone acetylation/methylation, have also been demonstrated to play an important role in regulating the differentiation and function of T cells. In CD4^+^ T cells, histone acetylation at the lineage-characteristic cytokine loci *Ifng* and *Il4* is crucial for Th1 and Th2 fate commitment [8, 9]. Likewise, genes involved in the effector function of CD8^+^ T cells, such as *Ifng*, *Eomes* and *Prf1*, acquire high abundance of histone acetylation during the differentiation of CD8^+^ T_N_ into T_M_ cells [10–12]. Moreover, a failure to undergo epigenetic reprogramming at effector gene loci contributes to poor functionality of T_M_ cells that are generated in the absence of CD4^+^ T cell help or during chronic virus infection [12, 13]. These studies have established a close link between histone acetylation and T cell differentiation.

The epigenetic landscapes in T cells are routinely studied in a gene-by-gene manner. For a handful set of genes that are functionally critical in T cells, covalent modifications to histones can be analyzed by chromatin immunoprecipitation (ChIP), followed by quantitative PCR with locus-specific primers [10–12, 14]. Epigenetic modifications can also be determined on genome-wide scale by coupling ChIP with high-throughput sequencing (ChIP-Seq) [15]. Although these techniques have provided valuable information on the T cell epigenome, they are generally performed using a large number of purified cells. However, it is challenging to obtain adequate amount of cells for rare populations, such as pathogen- and tumor-specific T cells. More recently, DiSpirito et al. [16] reported a flow cytometry-based assay to analyze histone modifications in combination with lineage-identifying markers on per-cell level, and they have identified a correlation between per-cell histone acetylation and the functionality of murine T cells. Whether the same role is applicable in human T cells have not been tested.

In this study, we employed a flow cytometric assay to examine histone acetylation in human T cell subsets on single-cell basis. The data showed that T cell activation with various stimulators led to elevated levels of histone acetylation. When making comparisons among different subsets, we found that histone acetylation was enriched in T_EMRA_ cells and positively correlated with effector cytokine production. Moreover, there is an inverse correlation between histone acetylation and expression of TCF-1, a critical transcription factor to maintain stem cell-like attributes in T cells [17, 18]. Finally, pharmaceutical inhibition of histone acetylation reduced the production of effector cytokines and preserved a less differentiated state in T cells expanded under a procedure for manufacturing chimeric antigen receptor (CAR) T cells. Together, our data indicate that terminally differentiated T cells are marked by hyper-histone acetylation in humans, which provides a significant cue for improving the efficacy of immunotherapy.

## Methods

### Isolation of mononuclear cells and treatments

Isolation of peripheral blood mononuclear cells (PBMCs) from healthy volunteers (25–55 years old, informed consent were obtained from all volunteers) was previously described [19]. In brief, PBMCs were prepared by density gradient centrifugation with Ficoll-Paque™ mononuclear cell preparation solution (GE Healthcare). Cells were cultured in R10 medium (RPMI 1640 + 10% fetal bovine serum) (Gibco) at a density of 1 × 10^6^ cells/mL, left unstimulated, or stimulated with phorbol 12-myristate 13-acetate (PMA) (50 ng/mL) plus ionomycin (I) (1 μg/mL) (Sigma Aldrich), anti-CD3 (50 ng/mL)/CD28 (100 ng/mL) monoclonal antibodies (mAbs) (Miltenyi Biotec.) or CMV pp65_(495–503)_ peptide (NLVPMVATV) (1 μg/mL) for 4–6 h. To expand T cells, PBMCs were stimulated with anti-CD3/CD28 mAbs in combination with rhIL-2 (PeproTech) (200 IU/mL) for the first 2 days, with or without 5 µM C646 (Absin). After that, cells were replated in R10 medium supplemented with rhIL-2 alone, and the medium was replenished every three days. In some experiments, CD8^+^ T cells were enriched after treatments by magnetic cell sorting (StemCell).

### Flow cytometry

Flow cytometric analysis for histone modification was described previously [16]. Briefly, cells were first incubated with Live/Dead Aqua Dye (Invitrogen) and then stained with antibody cocktails specific to cell surface markers (CD3, CD8, CD45RA, CD27, CD127, KLRG1, CD122 and CD95) (BioLegend). After that, cells were fixed and permeabilized with Foxp3/Transcription factor staining buffer (eBiosciences) followed by incubating with rabbit mAbs to acetyl-histone H3 (acH3) (K27, K9), acH4 (K16) and TCF-1 (Cell Signaling Tech.).

Intracellular cytokine staining (ICS) was performed as described previously [13]. In brief, cells were stimulated as indicated; brefeldin A (BD Biosciences) was added during the last 4 h of stimulation. Cells were first stained with viability dye, followed by staining with surface antigens. After permeabilization, cells were incubated with mAbs to IFN-γ, TNF-α and IL-2 (BioLegend) in combination with anti-acH3 (K27) and TCF-1. Data were acquired on FACS Canto II or Celesta flow cytometer (BD Biosciences) and analyzed with FlowJo software (Treestar).

### Western blot

Magnetically purified CD8^+^ T cells were lysed with RIPA buffer. Cell lysates were separated by SDS-PAGE and then transferred onto a nitrocellulose filter membrane (Millipore). Membranes were then subjected to immuno-blotting with an antibody specific to acH3 (K27); histone H3 (Cell Signaling Tech.) was used as loading control. The HRP signal was visualized with chemiluminescent HRP substrate (Millipore), and images were acquired using ChemiDoc™ XRS^+^ system (Bio-Rad).

### Confocal microscopy

Anti-CD3/CD28 stimulated or unstimulated CD8^+^ T cells were attached to poly-l-lysine treated microslides, fixed with 4% paraformaldehyde, and permeabilized with 0.1% Triton X-100. Cells were stained with a mouse mAb to human CD3 (Abcam), followed by staining with rabbit anti-mouse IgG-rhodamine and Alexa Fluor^®^ 488 conjugated anti-acH3 (K27). After washing, cells were incubated with an antifade solution containing DAPI, images were acquired using a Nikon A1^+^ R confocal microscope (Nikon).

### ELISA

Commercial kits for measuring IFN-γ and TNF-α concentrations in cell culture supernatants were purchased from R&D Systems (R&D Systems Inc.), and measurement of cytokines was carried out per the manufacturer’s instructions.

### Chromatin immunoprecipitation (ChIP)

The ChIP protocol was also described in the previous study [11]. Briefly, after indicated treatments, CD8^+^ T cells were crosslinked with 1% formaldehyde (37 °C for 10 min) and chromatin was sonicated into 200–1000 bp fragments, then immunoprecipitated with 5 μg of anti-acH3 using an EZ ChIP™ Chromatin Immunoprecipitation Kit (Upstate Biotech.) according to the manufacturer’s manual. Immunoprecipitated DNA was quantified by qPCR with the following primers: *IFNG* Pro F, 5′ TGGGTCTGTCTCATCGTCAA 3′, *IFNG* Pro R, 5′ CCTCCTCTGGCTGCTGGTAT 3′; *TNF* Pro F, 5′ CCAGGGTCCTACACACAAATCAGTC 3′, and *TNF* Pro R, 5′ CATTCAACCAGCGGAAAACTTC 3′.

### Quantitative RT-PCR

Measurement of gene expression by quantitative PCR was described previously [19]. Briefly, after indicated treatments, CD8^+^ T cells were magnetically purified. RNA was extracted using Trizol Reagent (Invitrogen); cDNA was synthesized using PrimeScript RT reagents (Takara Bio Inc.). mRNA expression was quantified with a 7500 Fast Real-Time PCR cycler (Applied Biosystems) using gene-specific primers, and primer sequences can be found in Additional file 1: Table 1.

### Statistical analysis

Summary values were presented as violin plots or mean ± SEM. Statistical differences between two groups were determined by Student two-tailed *t* test, or one-way ANOVA for comparison of multiple groups using GraphPad Prism software (GraphPad), and only *p* value < 0.05 was considered as statistically significant.

## Results

### T cell activation increases per-cell histone acetylation

**Fig. 1:**
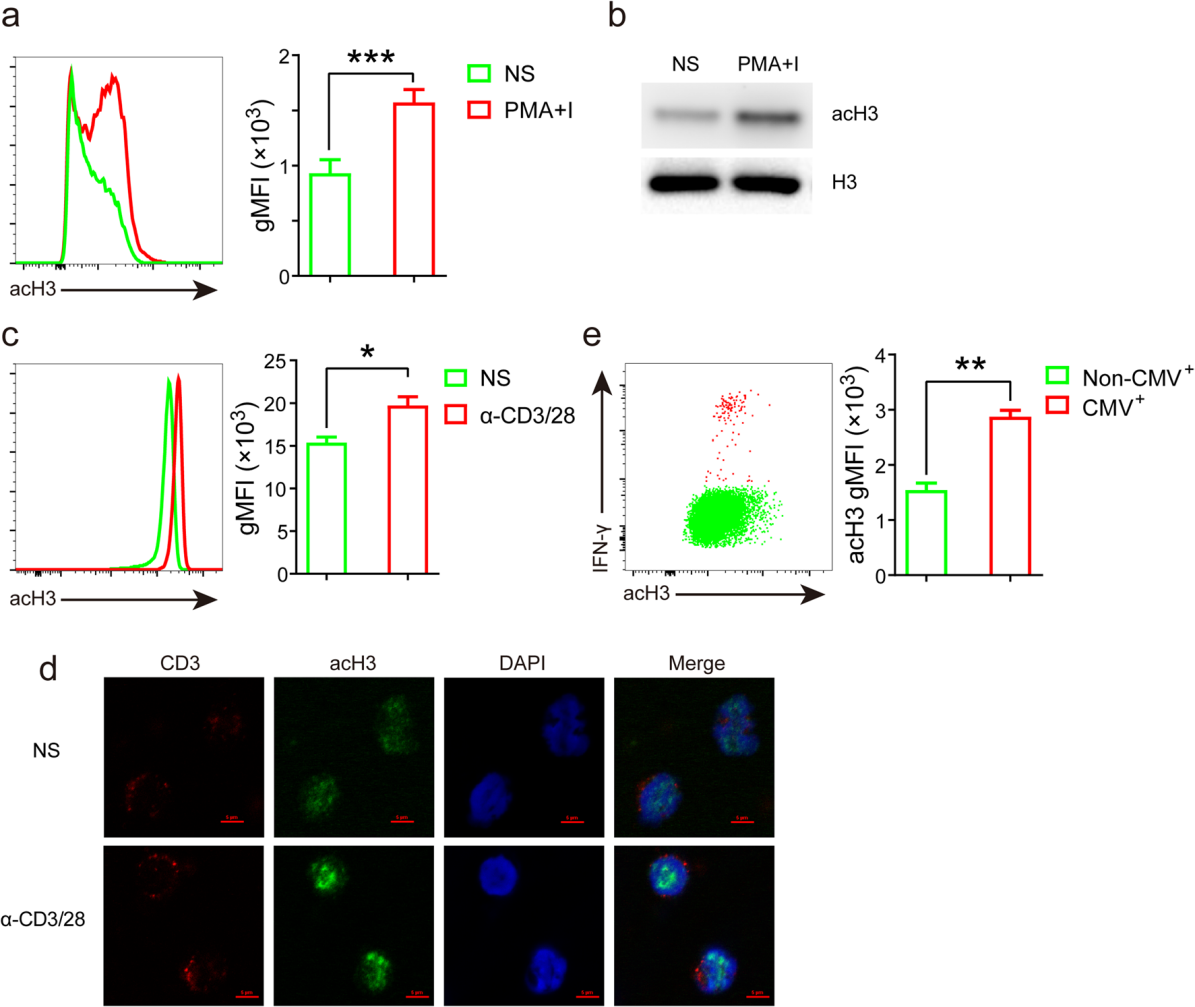
T cell activation led to elevated histone acetylation. **a** PBMCs (*N* = 37) were stimulated with/without PMA + I for 4 h; per-cell histone H3 (K27) acetylation (acH3) was measured by flow cytometry. Representative histograms (left) and geometric mean fluorescent intensity (gMFI) values (right) of acH3 in CD8^+^ T cells were shown. **b** PBMCs were treated as in (**a**) and then magnetically purified for CD8^+^ T cells, acH3 in cell lysates was visualized by western blot, total histone H3 served as loading control. **c** PBMCs (*N* = 13) were stimulated with/without anti-CD3 /CD28 mAbs for 4 h, acH3 was measured as in (**a**), shown were representative histograms (left) and summary results of gMFI (right). **d** Cells were treated as described in (**c**), and then magnetically purified for CD8^+^ T cells, expression of CD3 and acH3 were analyzed by confocal microscopy. Scale bars represent 5 μM. **e** PBMCs from HLA-A2^+^ individuals (*N* = 12) were stimulated with the CMV pp65 _(495–503)_ peptide in the presence of brefeldin A for 4 h; measurement of acH3 in CMV-specific (IFN-γ^+^) and non-specific (IFN-γ^−^) T cells was the same as in (**a**). Dot-plots of acH3 versus IFN-γ (left) and summary of acH3 gMFI (right) were shown. Experiments in (**b**) and (**d**) were repeated 5 and 3 independent times, respectively. *, *p* < 0.05; **, *p* < 0.01; ***, *p* < 0.001

T cell activation is required to execute effector functions in immune responses to both exogenous and endogenous antigens [20]. Therefore, we first assessed alterations in histone acetylation induced by T cell activation. PBMCs were stimulated with PMA + I for 5 h; histone H3 (K27) acetylation level in CD8^+^ T cells was then measured by flow cytometry. The data showed that per-cell histone acetylation was significantly higher in activated cells in comparison with non-activated cells (Fig. 1a). We also performed western blot to test the level of histone acetylation between non-activated and activated T cells using lysates from purified CD8^+^ T cells that were treated with/without PMA + I. Consistent with data validated by flow cytometry, we found that histone acetylation level was much higher in PMA + I stimulated cells than unstimulated cells (Fig. 1b). Activation of T cells by PMA + I typically bypasses proximal TCR-mediated signaling events [21]. To determine the impact of TCR engagement on histone acetylation, we stimulated cells with anti-CD3/CD28 mAbs. The results showed that histone acetylation in CD8^+^ T cells was also increased in the presence of anti-CD3/CD28 stimulation compared with non-stimulation (Fig. 1c). Similar to the FACS data, increased histone acetylation was also observed by confocal microscopy in anti-CD3/CD28 stimulated cells (Fig. 1d). Finally, we examined changes in histone acetylation in response to antigenic stimulation. To this end, PBMCs from HLA-A2^+^ individuals were stimulated with the CMV pp65 _(495–503)_ peptide, and antigen (Ag)-specific T cell responses were identified by IFN-γ production (Fig. 1e, left). In this assay, per-cell histone acetylation was much higher in IFN-γ producers than cells that were not secreting this cytokine, suggesting that antigenic stimulation also led to increased histone acetylation (Fig. 1e, right). Analogous to CD8^+^ T cells, per-cell histone acetylation was also elevated in CD4^+^ T cells after activation (data not shown). Together, both Ag-specific and broad T cell activation is accompanied with an up-regulation in histone H3 acetylation.

### T_EMRA_ cells display high level of per-cell histone acetylation

**Fig. 2:**
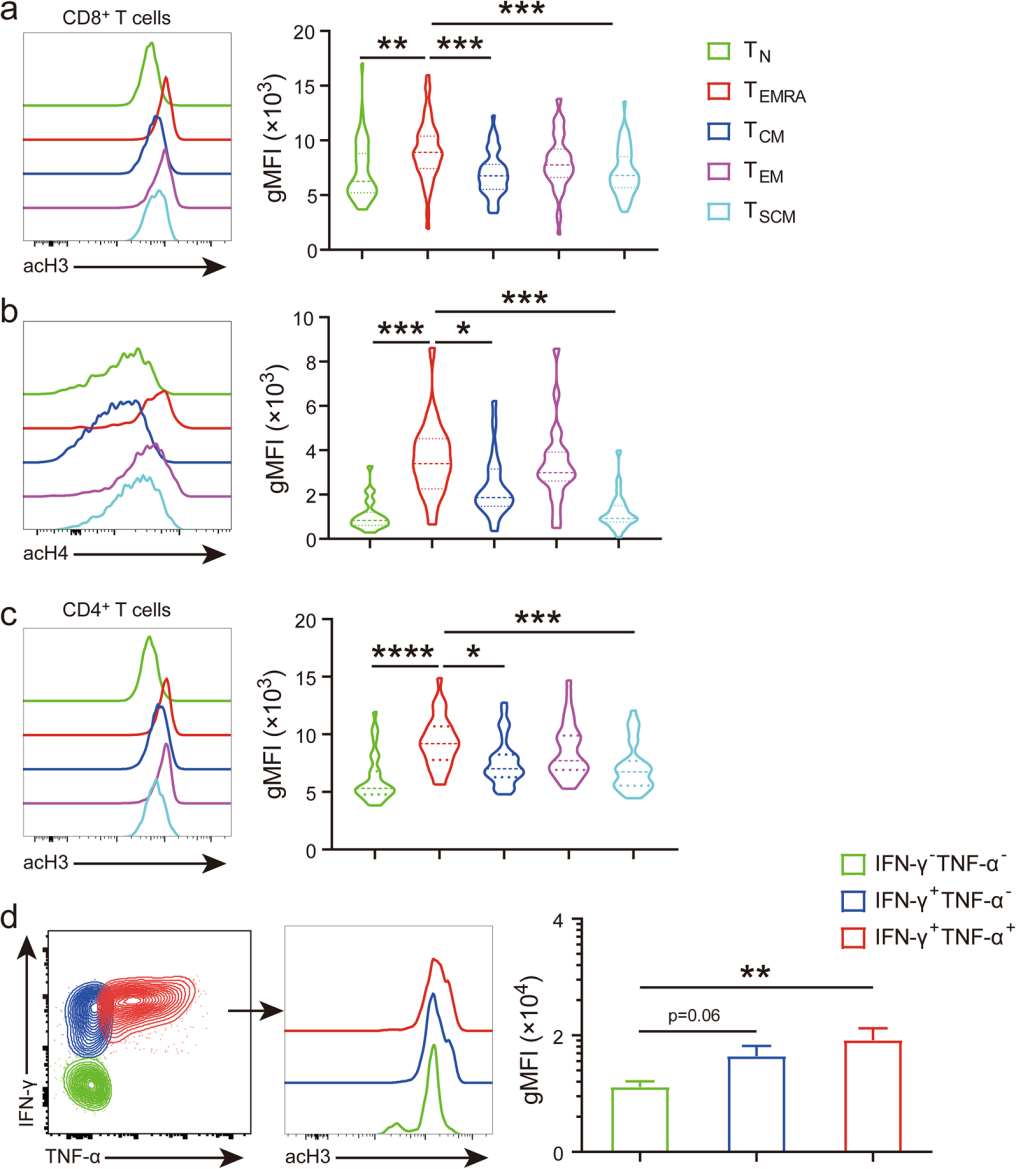
Per-cell histone acetylation was enriched in terminally differentiated human peripheral T cell subsets. **a**–**c**. Single-cell histone acetylation was stained in combination with surface markers to make comparisons among individual T cell subsets, representative histograms (left) and violin plots (right) summarizing gMFI of acH3 (**a**), acH4 (**b**) in CD8^+^, or acH3 in CD4^+^ (CD3^+^CD8^−^), (**c**) T_N_, T_EMRA_, T_CM_, T_EM_, and T_SCM_ were shown. **d** PBMCs were stimulated with PMA + I for 4 h in the presence of brefeldin A, ICS was performed to detect cytokine production (left), representative histograms (middle) and statistical results (right) of acH3 in IFN-γ^+^ TNF-α^+^, IFN-γ^+^ TNF-α^−^ and IFN-γ^−^ TNF-α^−^ CD8^+^ T cells were presented. Summary results derived from 51 individuals in (**a** & **c**), 23 individuals in (**b**), and 13 independent experiments in (**d**). *, *p* < 0.05; **, *p* < 0.01; ***, *p* < 0.001, ****, *p* < 0.0001

Epigenetic modifications have been linked to T cell differentiation and function [22, 23]. To test whether there is a correlation between histone modification and cellular identity globally, we compared histone acetylation in different subsets of T cells that were distinguished through the expression of several surface markers [6] (Additional file 2: Fig. 1). Among all subsets of CD8^+^ T cells, T_EMRA_ cells showed the highest level of histone acetylation at all three residues examined (H3K9, K27 and H4K16) on per-cell basis (Fig. 2a, b, Additional file 3: Fig. 2). Similar results were observed in CD4^+^ T cells, with T_EMRA_ cells possessing the highest per-cell histone H3 acetylation (Fig. 2c). Notably, aside from T_EMRA_ cells, histone acetylation was higher in T_EM_ cells than the rest of subsets (Fig. 2a–c, Additional file 3: Fig. 2). TEMRA cells were characterized by robust expression of genes that are important for the effector function of T cells [24]. To identify the functional relevance of histone acetylation, we performed intracellular cytokine staining (ICS) to analyze per-cell histone H3 acetylation in various effector cytokine producing CD8^+^ T cells following PMA + I stimulation. The results showed that the abundance of histone acetylation progressively decreased from cytokine co-producers (IFN-γ^+^ TNF-α^+^) to mono-producers (IFN-γ^+^ TNF-α^−^) and non-producers (IFN-γ^−^ TNF-α^−^) (Fig. 2d). Together, these data suggest that hyper-histone acetylation is a marker for terminally differentiated T cells with potent effector functions.

### Histone acetylation inversely correlates with TCF-1 expression

**Fig. 3:**
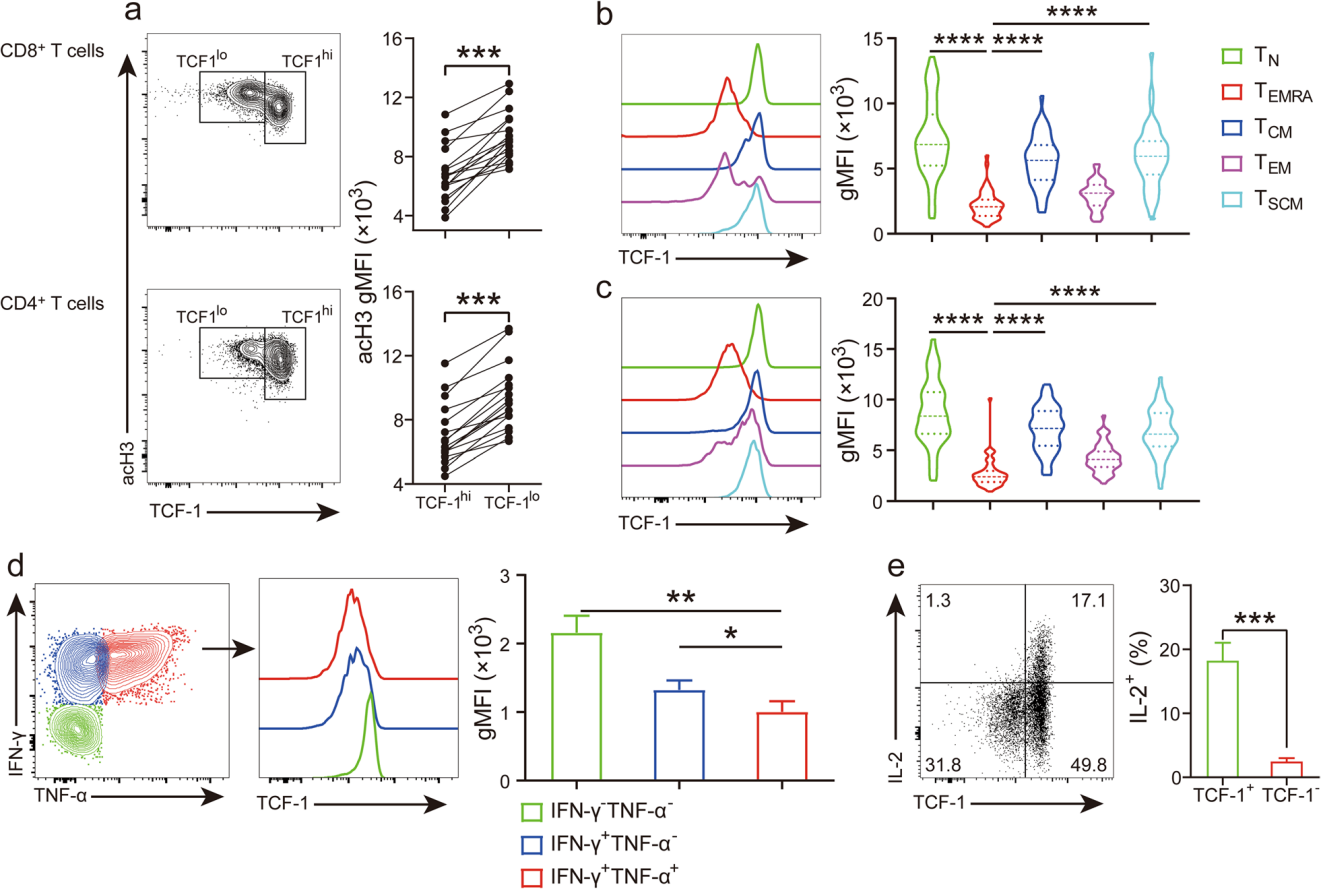
TCF-1 expression correlated with early differentiation state and hypo-effector function in human peripheral T cells. **a**–**c** Per-cell histone acetylation was stained together with TCF-1 and surface markers. **a** Representative dot-plots of acH3 versus TCF-1 (Left), and statistical analysis (*N* = 17) (right) of acH3 gMFI between TCF-1^hi^ and TCF-1^lo^ CD8^+^ (top) and CD4^+^ (CD3^+^CD8^−^) (bottom) T cells. acH3 gMFIs of TCF-1^hi^ and TCF-1^lo^ cells derived from the same donor were connected. **b** &** c** Histograms and summary results (*N* = 51) of TCF-1 expression in CD8^+^ (b) and CD4^+^ (CD3^+^CD8^−^) (c) T cell subsets. **d** &** e** ICS was performed the same as mentioned in Fig. 2d. **d** Representative images of effector cytokine production (left), TCF-1 expression in IFN-γ^+^ TNF-α^+^, IFN-γ^+^ TNF-α^−^ and IFN-γ^−^ TNF-α^−^ CD8^+^ T cells (middle), and a summary graph (*N* = 13) of TCF-1 gMFI in the three aforementioned T cell populations (right). **e** Representative dot-plots of IL-2 production versus TCF-1 expression (left) in CD8^+^ T cells, and statistical analysis (*N* = 13) on the frequency of IL-2^+^ cells in TCF-1^+^ and TCF-1^−^ subpopulations (right)

T cells are heterogeneous in their stem cell-like properties [6]. Since we have shown that histone acetylation is enriched in more terminally differentiated T cell subsets, we have special interests in understanding epigenetic mechanisms that modulate the stemness of T cells. For this aim, we measured per-cell histone acetylation in parallel with the expression of TCF-1, a transcription factor essential for retention of stemness [17]. The results showed that cells expressing high levels of TCF-1 were hypo-acetylated within total CD8^+^ and CD4^+^ T cell populations (Fig. 3a). We further compared TCF-1 expression in individual T cell subsets. As expected, the expression of TCF-1 in less differentiated subsets (including T_N_, T_CM_ and T_SCM_) was remarkably higher than T_EMRA_ and T_EM_ for both CD8^+^ and CD4^+^ T cells, which was in contrary to histone acetylation levels (Figs. 2, c and 3b, c). We also compared TCF-1 expression per effector cytokine production. Again, unlike histone acetylation levels, TCF-1 expression was the highest in CD8^+^ T cells producing none of the two effector cytokines, followed by cells producing IFN-γ^+^ alone and co-producers of IFN-γ^+^ and TNF-α^+^ (Fig. 3d). Reduced production of effector cytokines in TCF-1^hi^ cells might result from insufficient activation. To test this possibility, we examined IL-2 production, a cytokine that is typically produced by less differentiated cells [25]. The results revealed that ~ 15% of CD8^+^ T cells were producing IL-2. More importantly, the majority of IL-2 were produced by TCF-1^hi^ cells (Fig. 3e). Therefore, these data suggest a negative correlation between global histone acetylation and stem cell-like properties in T cells.

### Inhibition of histone acetylation decreases effector cytokine production and preserves stem cell-like attributes in T cells

Our previous data have demonstrated that hyper-histone acetylation is associated with strong effector function and poor stemness. Therefore, we hypothesized that inhibition of histone acetylation can reduce the expression of effector molecules and enhance stem cell-like features. To test our hypothesis, we stimulated T cells with anti-CD3/CD28 mAbs plus rhIL-2, which is analogous to the classical procedure for expansion of CAR T cells [26] and treated cells with C646, a small molecule inhibitor of p300/CBP histone acetyltransferases (HATs) [27]. We first tried different concentrations of this chemical inhibitor to test the optimal dose for inhibition. The results showed that both 5 and 25 μM of C646 potently inhibited histone acetylation to a similar extent (Additional file 4: Fig. 3a). However, treatment with high concentration of C646 resulted in a marked reduction in cell viability (Additional file 4: Fig. 3b); we thus chose 5 μM of C646 for further study.

**Fig. 4:**
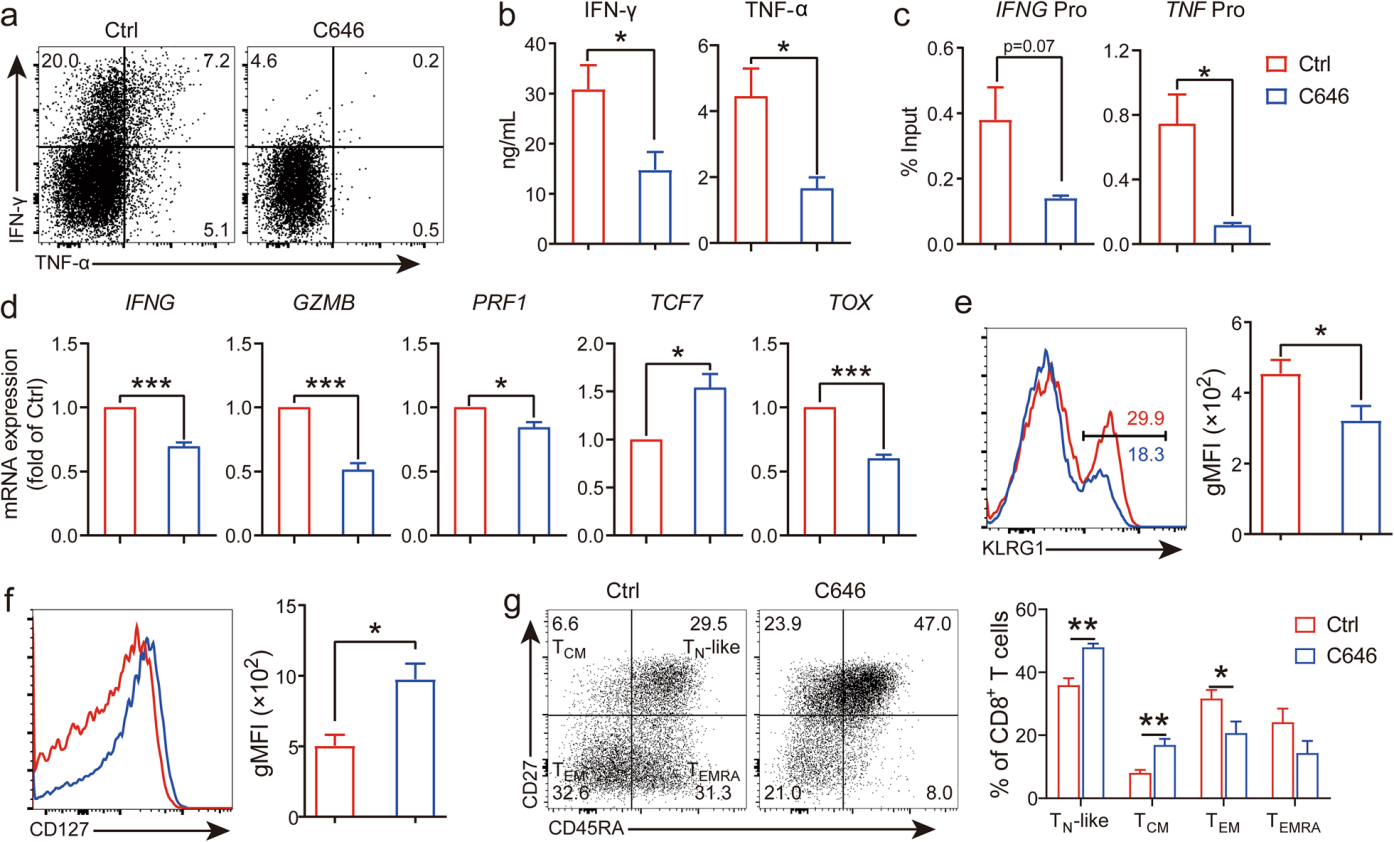
Inhibition of p300/CBP histone acetyltransferases reduced effector cytokine production and prevented terminal differentiation. PBMCs were stimulated with anti-CD3/CD28 mAbs plus rhIL-2 (200 IU/mL) for 48 h, with or without C646 (5 µM). **a** Brefeldin A was added during the last 4 h of stimulation; production of IFN-γ^+^ and TNF-α^+^ in C646-treated or untreated CD8^+^ T cells was measured by ICS. **b** IFN-γ^+^ and TNF-α concentrations in culture supernatants of PBMCs that were treated with/without C646 were detected by ELISA. **c** acH3 abundance at the promoter region of *IFNG* and *TNF* loci in C646-treated or untreated CD8^+^ T cells was assessed by ChIP. **d** mRNA expressions of *IFNG*, *GZMB*, *PRF1*, *TCF7* and *TOX* in C646-treated or untreated CD8^+^ T cells as determined by quantitative PCR, data were normalized to C646 untreated group. **e** &** f** Expression of KLRG1 (e) and CD127 (f) with/without C646 treatment, representative histograms (left) and summary results of gMFI (right) were shown. **g** Representative dot-plots of T cell subset compositions (left), and statistical presentation of individual T cell subsets between C646-treated and non-treated CD8^+^ T cells after 8-day expansion (right). All experiments were repeated three to five independent times with 1–3 individuals each time

Next, we tested whether alterations in histone acetylation impacted the effector function of T cells. We found that the fraction of cytokine producing CD8^+^ T cells remarkably decreased in the presence of C646 treatment (Fig. 4a). Consistently, the amount of IFN-γ and TNF-α in culture supernatants of C646-treated cells was significantly lower than vehicle-treated group (Fig. 4b). Reduced production of these two cytokines was associated with lower abundance of histone H3 acetylation at the promoter region of both gene loci, as determined by ChIP (Fig. 4c). To gain more insights into genes affected by C646 treatment, we performed quantitative PCR to analyze the mRNA level of a set of genes, which play critical roles in the effector function, stemness and exhaustion of CD8^+^ T cells. The results showed that key effector genes, including *IFNG*, *GZMB* and *PRF1*, were down-regulated by C646 treatment (Fig. 4d). Interestingly, *TCF7* (the gene encoding TCF-1) was up-regulated by C646 treatment, whereas the expression level of *TOX*, which is a newly identified regulator for T cell exhaustion [28], was decreased in the presence of C646 treatment (Fig. 4d). We also detected the expression of two surface markers, KLRG1 and CD127, which are preferentially expressed on terminal effectors and bona fide memory T cells, respectively [29]. In accordance with decreased production of effector molecules, KLRG1^hi^ cells were much fewer following C646 treatment (Fig. 4e). By contrast, expression of CD127 was elevated when treated with C646 (Fig. 4f). Decreased expression of effector genes could be a consequence of activation differences in C646-treated cells. To test this possibility, we examined the expression of IL-2 and CD69 (a marker of T cell activation) [30] and analyzed the expansion rates between C646-treated and non-treated CD8^+^ T cells. The results showed that IL-2 was similarly expressed by these two groups of cells, at both protein (Additional file 5: Fig. 4a) and mRNA (Additional file 5: Fig. 4b) levels. There were no significant differences in the expression of CD69 between C646-treated and untreated cells (Additional file 5: Fig. 4c). In consistent with these data, CD8^+^ T cell expansion was minimally impacted by C646 treatment at day 2 and day 8 during cell culture (Additional file 5: Fig. 4d). Finally, we examined the subset composition of T cells in the presence/absence of C646 treatment after expansion. The results showed that the proportion of T_CM_ and T_N_-like cells was increased, whereas the frequency of T_EM_ cells was decreased after C646 treatment (Fig. 4g). Interestingly, these T_CM_ and T_N_-like cells exhibited lower level of histone acetylation but higher degree of stemness (TCF-1) than T_EM_ cells (Figs. 2a, 3b), indicating C646 treatment upregulated stem cell-like T cell subsets by inhibition of histone acetylation. Together, these data suggest that suppression of histone acetylation could restrain the effector function but maintain the stemness of T cells.

## Discussion

Human peripheral T cell repertoire is highly heterogeneous, containing at least five subsets, which are phenotypically distinct from each other [31]. Different features of T cells are partially elucidated by their transcriptomic signatures, which are largely encoded epigenetically, but the underlying mechanisms remain poorly understood [22]. To solve this knowledge gap, we described for the first time that per-cell histone acetylation was correlated with the differentiation state and effector function in human T cells. Other histone modifications, such as histone methylation, also play a critical role in regulating T cell differentiation and function [15]; it will be of interest to generate a comprehensive map by combining different histone modifications at single-cell level to illustrate the epigenome of human T cells in future study.

In our study, per-cell histone acetylation was found to be enriched in human T cell subsets close to terminal differentiation, like T_EMRA_ and T_EM_ cells. These results are not identical to a previous report in mice, where they presented that histone acetylation is a marker of functional T_M_ cells [16]. These observations possibly reveal specie-specific differences in T cell epigenomics. Actually, many aspects in T cell biology have also been demonstrated to be different between human and mice. For instance, replenishing the naïve T cell pool in mice largely relies on thymic output, whereas in humans maintenance of naïve T cells depends on renewal in the periphery [32]. There are also considerable differences in the distribution of tissue-resident T_M_ cells (T_RM_) between these two species; specifically, T_RM_ cells are more abundant in lymphoid organs in humans in comparison with mice [33]. Our data together with previous studies highlight the importance to study human T cells, which are closer to translation in clinical, in conjunction with murine models, which offer many experimental advantages.

Progressive differentiation of T_N_ cells into T_M_ cells gives rise to heterogeneous subpopulations representing various differentiation stages. Among them, T_EMRA_ and T_EM_ cells are thought to be committed to terminal differentiation, whereas T_CM_ and T_SCM_ cells display enhanced self-renewal and long-term persistence [4, 6, 24]. Here we validated the elevated expression of the critical transcription factor TCF-1 in human peripheral T cell subsets with enhanced stem cell-like properties and also demonstrated a negative correlation between TCF-1 expression and global histone acetylation. A population of TCF-1-expressing T cells have also been identified within exhausted T cells induced by chronic virus infection [17] and in human tumor-infiltrating lymphocytes (TILs) [34]. Existence of this TCF-1^+^ T cell population with stem cell-like features contributes to better treatment responses to immune checkpoint inhibitors and host survival [18]. Regarding the current finding of an inverse correlation between per-cell histone acetylation and stem cell-like attributes in T cells, targeting this important epigenetic modification could be a promising strategy to enhance stemness of T cells and promote the immune control of chronic pathogens as well as tumors.

Our results will also have important implications in adoptive cellular immunotherapy (ACI). The emergence of CAR-T therapy represents one of the most prominent advances in the treatment of cancers in the past two decades. Although great success has been achieved in treating hematopoietic malignancies [26, 35], very rare patients with solid tumors acquire durable benefits from this therapy [36]. One reason accounting for the failure of CAR-T therapy is the differentiation of T cells into terminal effectors during expansion, which lack stemness and are incapable to persist in vivo after adoptive transfer [37, 38]. Our data showed that chemical inhibition of histone acetylation with C646 during T cell expansion limited effector functions of T cells, preserving them in less differentiated state. Interestingly, this small molecule inhibitor did not show negative impacts on the activation and expansion of T cells, indicating a great translational potential in ACI. In fact, inactivation of TET2 and deletion of DNMT3A have proven to be efficient in potentiating anti-tumor immunity by adoptively transferred cells [39, 40]. These important findings together with the current study provide fundamental basis for epigenetically reprogramming T cells to improve therapeutic efficacy of ACI.

In summary, we validated the use of a flow cytometric assay in deciphering the epigenetic code in human T cells and identified a correlation between per-cell histone acetylation and T cell differentiation state, which suggests a new direction to enhance the efficacy of immunotherapy.

## Conclusions

Our results reveal a negative association between per-cell histone acetylation and the stem cell-like properties of human T cells. These findings provide a novel druggable target to enhance the stemness of T cells and improve the efficacy of immunotherapy.

### Supplementary Information

**Additional file 1: Table 1.** Primers sequences used for quantification of mRNA expression levels

**Additional file 2: Fig. 1.** Gate strategies used to classify human peripheral T cell subsets. Lymphocytes were first gated, singlets and live cells were gated subsequently. Within the live cell population, CD8^+^ and CD4^+^ T cells were gated based on the expression of CD3 and CD8. Both CD3^+^CD8^+^ and CD3^+^CD8^−^ (CD4^+^) T cell subsets were defined as following: T_N_-like, CD45RA^+^CD27^+^; T_CM_, CD45RA^−^CD27^+^; T_EM_, CD45RA^−^CD27^−^ and T_EMRA_, CD45RA^+^CD27^−^. T_N_-like cells were further divided into CD122^+^CD95^+^ T_SCM_ and CD122^+/−^CD95^−^ T_N_ subsets.

**Additional file 3: Fig. 2.** Histone H3 (K9) acetylation in CD8^+^ T cell subsets. Anti-histone H3 (K9) mAb was stained together with cell surface markers as described in Fig. 2a. Representative results of acH3 (K9) in CD8^+^ T_N_, T_EMRA_, T_CM_, T_EM_, and T_SCM_ cells among 23 individuals were shown by histograms (left), and the distribution of acH3 (K9) gMFI was summarized in violin plots (right).

**Additional file 4: Fig. 3.** Effects of C646 treatment on per-cell histone acetylation and cell viability. PBMCs were stimulated with anti-CD3/CD28 mAbs plus rhIL-2, and treated with 0, 5 or 25 µM C646 for 48 h. **a** Per-cell histone acetylation in CD8^+^ T cells after treatment with different concentrations of C646. **b** Fraction of viable lymphocytes in the presence/absence of C646 treatment. All experiments were repeated at least five independently times.

**Additional file 5: Fig. 4.** Impacts of C646 treatment on CD8^+^ T cell activation and expansion. PBMCs were stimulated with anti-CD3/CD28 mAbs plus rhIL-2, and treated with vehicle (Ctrl) or 5 µM C646 for 48 h. **a** Representative images (left) and summary graphs of IL-2 production (right) in non-stimulated (NS), Ctrl or C646 treated CD8^+^ T cells. **b** *IL-2* mRNA expression in magnetically purified NS, Ctrl or C646 treated CD8^+^ T cells. **c** Representative histogram (left) and summary results of CD69 expression (right) by NS, Ctrl or C646 treated CD8^+^ T cells. **d** Expansion folds for Ctrl or C646 treated CD8^+^ T cells after 2- or 8-day culture. Summary results are derived from 3 to 5 independent experiments.

## Data Availability

All data generated in this study are available from the corresponding author on reasonable request.
