# Supplementary material for: Per-cell histone acetylation is associated with terminal differentiation in human T cells
Source: Clin Epigenetics. 2024 Feb 6;16:21. doi: 10.1186/s13148-024-01634-w (PMC10845582; doi:10.1186/s13148-024-01634-w)
Supplement: Supplementary file 1 — Additional file 1: Table 1. Primers sequences used for quantification of mRNA expression levels [file 13148_2024_1634_MOESM1_ESM.docx]

Supplementary Table 1. Primers sequences used for quantification of mRNA expression levels

| Gene Symbol | Forward | Reverse |
| --- | --- | --- |
| *PRF1* | GTGGAGTGCCGCTTCTACAGTT-3 | TGCCGTAGTTGGAGATAAGCCT |
| *GZMB* | GGTGGCTTCCTGATACAAGACG | GGTCGGCTCCTGTTCTTTGAT |
| *IFNG* | AACTTCTTTGGCTTAATTCTC | GAGTTCCATTATCCGCTACATC |
| *TCF7* | CTGGCTTCTACTCCCTGAC | ACCAGAACCTAGCATCAAG |
| *TOX* | TATGAGCATGACAGAGCCGAG | GGAAGGAGGAGTAATTGGTGGA |
| *IL2* | AGAAGAACTCAAACCTCTGGAGGAA | CAATGGTTGCTGTCTCATCAGCATA |
| *GAPDH* | GAAGGTCGGAGTCAACGGAT | CCTGGAAGATGGTGATGGG |
