# Supplementary figures and images for: Per-cell histone acetylation is associated with terminal differentiation in human T cells
Source: Clin Epigenetics. 2024 Feb 6;16:21. doi: 10.1186/s13148-024-01634-w (PMC10845582; doi:10.1186/s13148-024-01634-w)

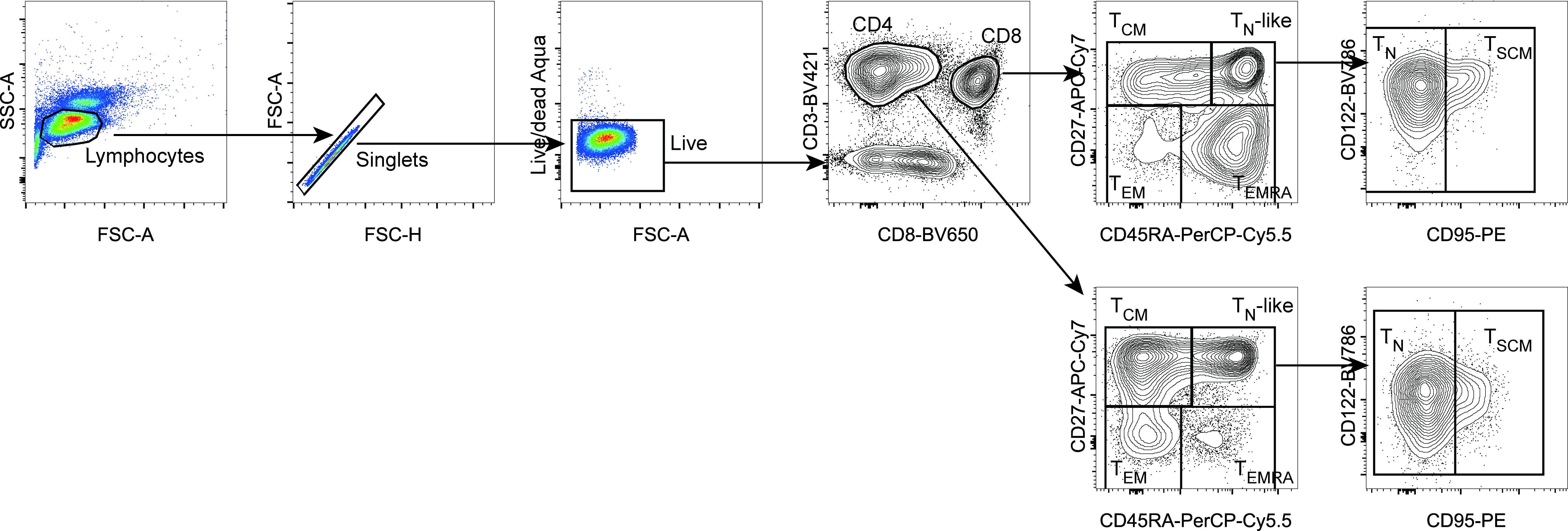

Supplement: Supplementary file 2 — Additional file 2: Fig. 1. Gate strategies used to classify human peripheral T cell subsets. Lymphocytes were first gated, singlets and live cells were gated subsequently. Within the live cell population, CD8+ and CD4+ T cells were gated based on the expression of CD3 and CD8. Both CD3+CD8+ and CD3+CD8− (CD4+) T cell subsets were defined as following: TN-like, CD45RA+CD27+; TCM, CD45RA−CD27+; TEM, CD45RA−CD27− and TEMRA, CD45RA+CD27−. TN-like cells were further divided into CD122+CD95+ TSCM and CD122+/−CD95− TN subsets. [file 13148_2024_1634_MOESM2_ESM.tif]

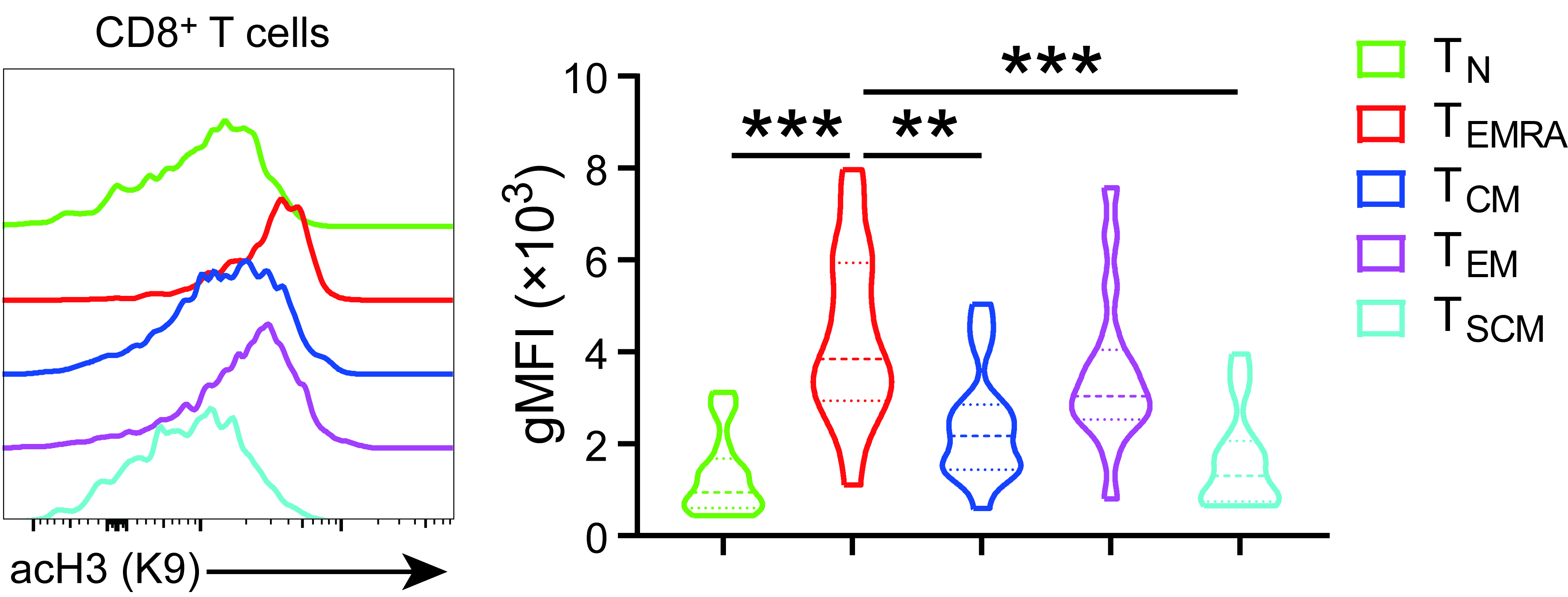

Supplement: Supplementary file 3 — Additional file 3: Fig. 2. Histone H3 (K9) acetylation in CD8+ T cell subsets. Anti-histone H3 (K9) mAb was stained together with cell surface markers as described in Fig. 2a. Representative results of acH3 (K9) in CD8+ TN, TEMRA, TCM, TEM, and TSCM cells among 23 individuals were shown by histograms (left), and the distribution of acH3 (K9) gMFI was summarized in violin plots (right). [file 13148_2024_1634_MOESM3_ESM.tif]

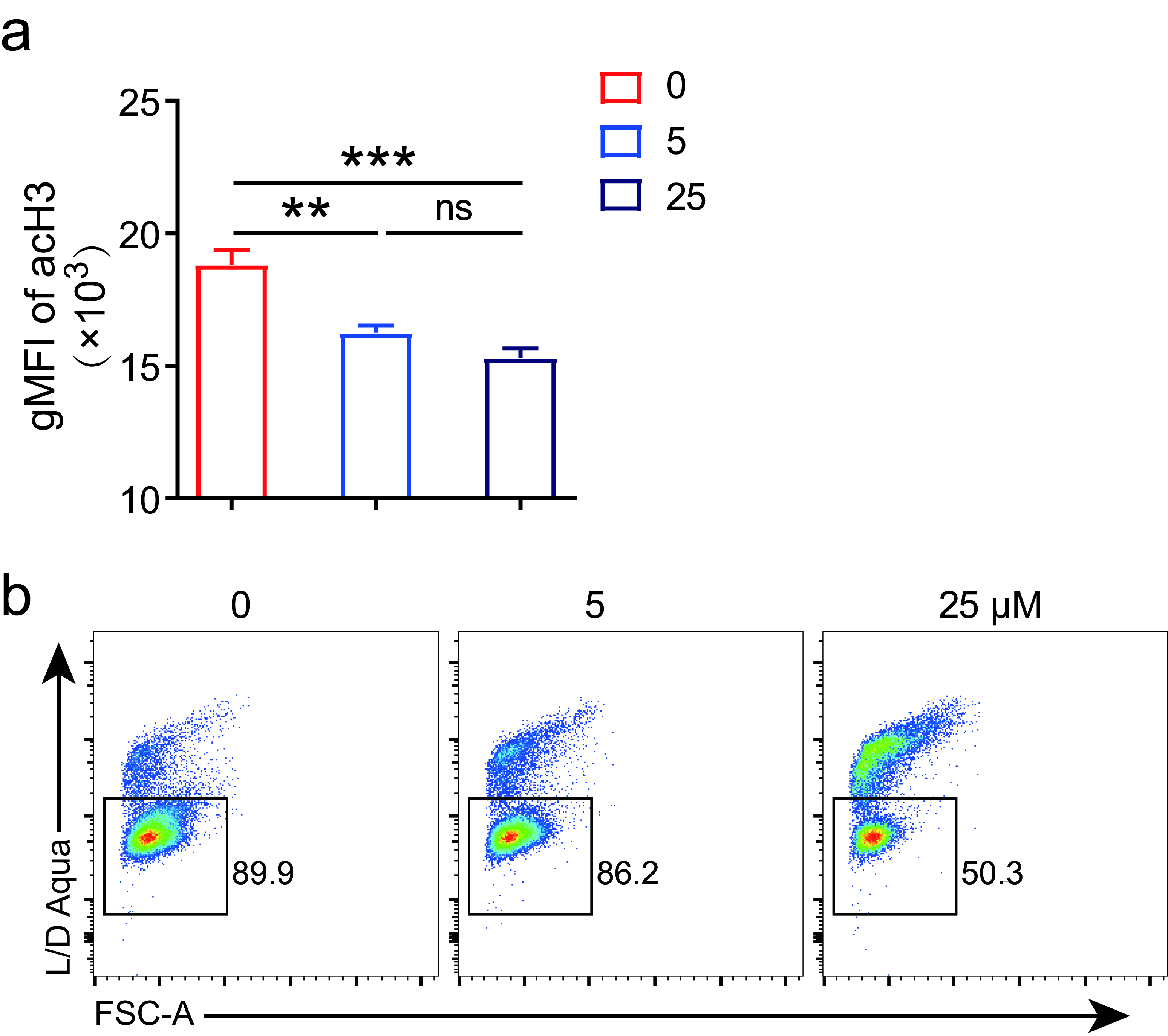

Supplement: Supplementary file 4 — Additional file 4: Fig. 3. Effects of C646 treatment on per-cell histone acetylation and cell viability. PBMCs were stimulated with anti-CD3/CD28 mAbs plus rhIL-2, and treated with 0, 5 or 25 µM C646 for 48 h. a Per-cell histone acetylation in CD8+ T cells after treatment with different concentrations of C646. b Fraction of viable lymphocytes in the presence/absence of C646 treatment. All experiments were repeated at least five independently times. [file 13148_2024_1634_MOESM4_ESM.tif]

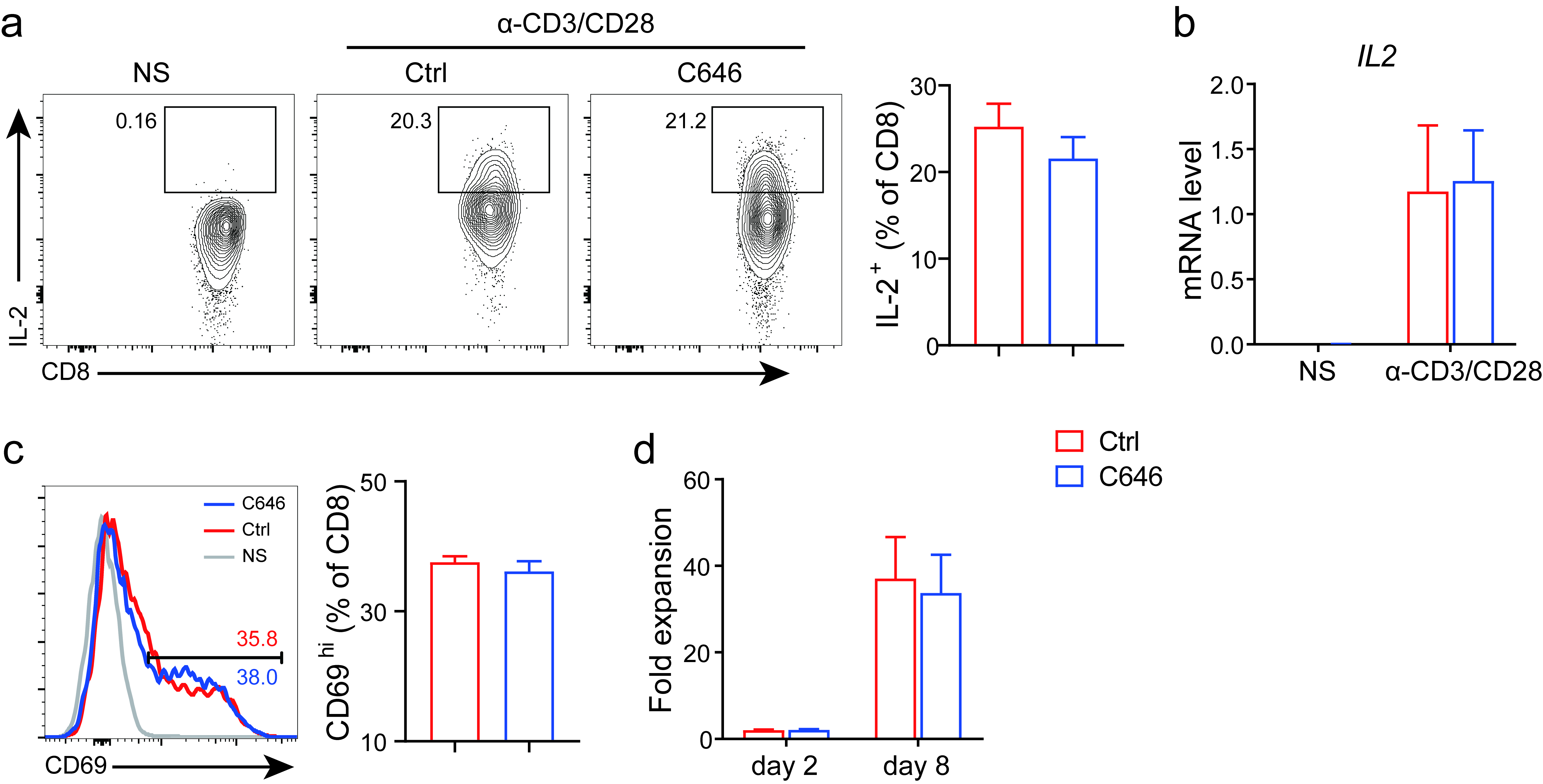

Supplement: Supplementary file 5 — Additional file 5: Fig. 4. Impacts of C646 treatment on CD8+ T cell activation and expansion. PBMCs were stimulated with anti-CD3/CD28 mAbs plus rhIL-2, and treated with vehicle (Ctrl) or 5 µM C646 for 48 h. a Representative images (left) and summary graphs of IL-2 production (right) in non-stimulated (NS), Ctrl or C646 treated CD8+ T cells. b IL-2 mRNA expression in magnetically purified NS, Ctrl or C646 treated CD8+ T cells. c Representative histogram (left) and summary results of CD69 expression (right) by NS, Ctrl or C646 treated CD8+ T cells. d Expansion folds for Ctrl or C646 treated CD8+ T cells after 2- or 8-day culture. Summary results are derived from 3 to 5 independent experiments. [file 13148_2024_1634_MOESM5_ESM.tif]
